# Effect of Feeding Palm Oil By-Products Based Diets on Muscle Fatty Acid Composition in Goats

**DOI:** 10.1371/journal.pone.0119756

**Published:** 2015-03-19

**Authors:** Abdelrahim Abubakr, Abdul Razak Alimon, Halimatun Yaakub, Norhani Abdullah, Michael Ivan

**Affiliations:** 1 Department of Animal Science, University Putra Malaysia, Serdang, Selangor, Malaysia; 2 Institute of Tropical Agriculture, University Putra Malaysia, Serdang, Selangor, Malaysia; 3 Department of Biochemistry, University Putra Malaysia, Serdang, Selangor, Malaysia; 4 Department of Animal Nutrition, University of Bahri,Khartoum, Sudan; Wageningen UR Livestock Research, NETHERLANDS

## Abstract

The present study aims to evaluate the effects of feeding palm oil by-products based diets on different muscle fatty acid profiles in goats. Thirty-two Cacang × Boer goats were randomly assigned to four dietary treatments: (1) control diet (CD), (2) 80% decanter cake diet (DCD), (3) 80% palm kernel cake diet (PKCD) and (4) CD plus 5% palm oil (PO) supplemented diet (CPOD). After 100 days of feeding, four goats from each group were slaughtered and *longissimus dorsi* (LD), *infraspinatus* (IS) and *biceps femoris* (BF) were sampled for analysis of fatty acids. Goats fed the PKCD had higher (*P*<0.05) concentration of lauric acid (C12:0) than those fed the other diets in all the muscles tested. Compared to the other diets, the concentrations of palmitic acid (C16:0) and stearic acid (C18:0) were lower (*P*<0.05) and that of linoleic acid (C18:2 *n*-6) was higher (*P*<0.05) in the muscles from goats fed the CD. It was concluded that palm kernel cake and decanter cake can be included in the diet of goats up to 80% with more beneficial than detrimental effects on the fatty acid profile of their meat.

## Introduction

Goats are important meat-producing animals in the tropics [1,2] and the goat’s meat (chevon) has been increasingly consumed around the world [3] especially in the developed countries, mainly because of its relatively low fat contents [4]. Also, compared to beef and sheep, the goat carcass tends to have more internal and less subcutaneous and intramuscular fat [5–7]. It has been reported that human nutrition and health were affected by the content and composition of intramuscular fat [8]. Saturated fatty acids (SFA) are associated with coronary diseases [9] whereas poly-unsaturated fatty acids (PUFA) might have health benefits for consumers [10]. Generally, in ruminant muscle lipids, the proportion of saturated fatty acids (SFA) are often higher [11] and the poly-unsaturated fatty acids (PUFA)/SFA ratio lower, because dietary unsaturated fat is hydrogenated in the rumen by the action of rumen microbes. While the muscle fatty acid profile in ruminants is less affected by the dietary fatty acids compared to non-ruminants, different type of diets can affect the fatty acid profile of the meat [7,12–17].

One of the major constrains in developing ruminant livestock industry in Malaysia is the difficulty to provide sufficient feed in terms of quantity and quality throughout the year [18]. In most humid tropics, goats usually feed on tree leaves or grasses [19]. Malaysian palm oil industry produces a huge amount of by-products annually, including crude palm oil (PO), oil palm frond, palm press fiber, palm kernel cake (PKC) and palm oil decanter cake (DC). These by-products represent an alternative, as they are readily available and represent sustainable feed resources for ruminants and other farm animals. Their inclusion in diets can be an effective measure in overcoming the lack of grazing pasture for small ruminants. Palm kernel cake and DC were tested as feed for dairy goats [20,21], lambs [22] and cattle [23]. More recently it has been reported that the PKC and DC based diets fed to goats result in increased fat deposition due to the relatively higher ether extract contents of PKC and DC [24]. The hypothesis of the present study was that PKC and DC inclusion in the diet of goats would also change the fatty acid composition of their muscles. Thus, the objective of the present experiment was to determine the effects of the dietary PKC, DC and PO on the fatty acid composition in muscles of goats.

## Material and Methods

### 2.1 Animal and diets

Muscle samples analyzed in the present study were collected at the end of a growth experiment conducted in the small ruminant unit, University Putra Malaysia. Results of the growth, nitrogen metabolism and carcass characteristics were described in details elsewhere [24]. Briefly, thirty two approximately 4–6 months old (average 5 months) Kajang-Boer crossbred male goats with initial body weight of 16.9 ± 0.4 kg were randomly divided into four groups of eight animals each. The goats were housed individually in wooden pens with slatted flooring in an open sheep barn raised above ground. After a three-week adaptation period, the goats were each randomly assigned to one of four dietary treatments in a 100-day experiment. The four treatments were (1) control diet (CD), (2) diet containing 80% DC (DCD), (3) diet containing 80% PKC (PKCD) and (4) diet containing PO (CPOD), where 5% PO replaced an equal amount of corn grain in the diet CD (Table 1). The diets were approximately iso-nitrogenous. The animals were fed the assigned diets as total mixed ration (TMR) *ad libitum* once a day at 08:00. Clean water was available *ad libitum* throughout the experiment. All goats were weighed monthly before morning feeding and their care and sacrifice were in accordance with the country standards; the experimental protocol was reviewed and approved by the University Putra Malaysia Animal Care and Use Committee.

**Table 1:** Ingredients (%) and chemical composition (% DM) of diets and chemical composition of their major ingredients (decanter cake, palm kernel cake).

Ingredients/Diets	PO	PKC	DC	CD	DCD	PKCD	CPOD
Rice straw				10	10	10	10
Corn grain				65	1	1	60
Soybean meal				22	6	6	22
Palm kernel cake				0	0	80	0
Decanter cake				0	80	0	0
Palm oil				0	0	0	5
Molasses				1.25	1.25	1.25	1.25
Urea				0.75	0.75	0.75	0.75
Salt				0.5	0.5	0.5	0.5
Dicalcium phosphate				0.5	0.5	0.5	0.5
Chemical composition							
Dry matter %		94.7	72.4	89.5	75.9	93.8	95.9
Organic matter		93.9	93.7	95.7	91.5	93.3	96.0
Crude protein		15.9	15.7	16.4	16.3	16.3	16.0
Ether extract		9.1	10.9	2.8	7.4	7.6	7.6
Ash		6.1	6.3	4.3	8.5	6.7	4.0
Neutral detergent fiber		72.3	45.6	19.7	45.6	67.0	19.1
Acid detergent fiber		47.6	17.2	10.2	19.8	44.1	10.0
Acid detergent lignin		17.3	13.8	1.1	6.1	14.5	1.1
Fatty acid composition (g/100g fatty acids)							
C12:0	1.69	52.13	0.47	NA	0.02	53.41	0.01
C14:0	0.58	15.38	0.88	NA	0.50	16.21	0.81
C16:0	49.64	8.65	38.98	9.87	40.35	4.26	45.53
C18:0	3.76	4.63	4.65	5.82	5.80	11.21	4.45
C18:1	35.40	15.64	43.77	29.43	38.76	7.80	40.64
C18:2	7.34	1.39	9.44	54.43	12.12	4.57	8.94
C18:3	1.08	1.77	1.38	0.51	2.34	2.44	0.22
C20:0	0.51	0.41	0.43	0.34	0.11	0.10	0.21

PKC, palm kernel cake; DC, decanter cake; CD, control diet; DCD, decanter cake diet; PKCD, palm kernel cake diet; CPOD, control plus 5% palm oil diet.

### 2.2. Slaughtering procedure and sampling

At the end of the feeding experiment, four animals from each group were selected randomly for slaughter after overnight fasting. The slaughtering was performed in accordance with the slaughtering procedure outlined in the Malaysian Standards (2004). Approximately 200 g of the *longissimus dorsi* (LD), *infraspinatus* (IS) and *biceps femoris* (BF) muscles were sampled from the left side of the carcass after overnight cooling at 4°C. All visible fat was removed from the meat surface, vacuum packed in polyethylene packs and stored at -80°C until analysis.

### 2.3. Proximate analysis of feed

The standard method of AOAC [25] was followed to determine the proximate chemical composition of feed samples. Samples of feed were dried in a forced-air oven for 24 h at 105°C to determine dry matter (DM). Nitrogen was determined by Kjeltec Auto Analyzer and then converted to crude protein (CP = N × 6.25). Ether extract (EE) was determined by extracting the sample with petroleum ether (40–60°C) using a Soxtec Auto Analyzer. Neutral detergent fiber (NDF), acid detergent fiber (ADF) and acid detergent lignin (ADL) were determined by the methods outlined by Van Soest et al. [26] and Robertson and Van Soest [27] without adding alpha amylase and sodium sulfite. Values for NDF and ADF are expressed inclusive of residual ash. Samples were ashed in a muffle furnace at 550°C for 4 h to determine the ash content. Each analysis was performed in triplicate.

### 2.4. Determination of fatty acid composition

The method described by Folch et al. [28] and modified by Rajion et al. [29] was used to extract total fatty acids from feed and muscle samples using chloroform/methanol 2:1 (v/v) containing butylated hydroxytoluene to prevent oxidation during sample preparation. Briefly, approximately 1 g of feed or muscle samples were homogenized in 40 ml chloroform/methanol solution in a 50 ml ground-glass extraction tube and the mixture was filtered. Normal saline solution (10 ml) was added to ease phase separation. Thereafter, the lower phase was collected in a round bottom flask, rotary evaporated at 70°C for 15–20 minutes and heneicosanoic acid (C21:0; Sigma Chemicals, St. Louis, MO, USA) was added as an internal standard to each sample. The extract of fatty acids was saponified and esterified according to the AOAC [25] to prepare fatty acid methyl esters (FAME). The prepared FAME was analyzed using gas chromatography (GC; Agilent 6890) equipped with an automatic sampler. A 100 m × 0.25 mm ID × 0.2 μm film thickness capillary column was used to separate the methyl esters, which were detected with a flame ionization detector (FID). The injection temperature was 250°C, and the column was programmed to run at 150°C for 2 min, warmed to 158°C at 1°C/min, held for 28 min, warmed to 220°C at 1°C/min and then held for 20 min to achieve a satisfactory separation. Nitrogen was the carrier gas with a flow rate at 1.2 ml/min, and split ratio of 1:20. The identification of individual FAME in the sample was achieved by matching the retention time with FAME standard mixture (Sigma Chemicals). The fatty acid concentrations are expressed as g/100 g of the sum of identified peaks measured in each sample.

### 2.5. Statistical analysis

Fatty acid profiles in different muscles were analyzed by one-way ANOVA as completely randomised design using GLM procedure of SAS [30]. Diets were used as treatment effect, with individual animal as the experimental unit. Treatment means were separated by Duncan multiple range test [31] at *P* < 0.05.

## Results

### 3.1. Fatty acid composition of *longissimus dorsi*

The concentration of C12:0 in LD was higher (*P*<0.05) for the PKCD than for the CD, whereas goats receiving the DCD and CPOD showed similar concentrations to that for the CD (Table 2). The concentration of C16:0 was higher (*P*<0.05) for the DCD and CPOD than for the CD, with no differences between the CD and PKCD. The concentration of C18:0 was higher (*P*<0.05) for the DCD and CPOD compared to the CD without any significant differences between the CD and PKCD. The concentration of C18:2 *n*-6 was highest (*P*<0.05) for the CD than for the other diets. The differences among the dietary treatments in concentration of the rest of individual fatty acids in the LD were not significant.

**Table 2:** Effect of dietary treatments on the fatty acid composition (g/100g of total fatty acids) of the *longissimus dorsi* muscle of goats.

Fatty acids	CD	DCD	PKCD	CPOD	SE	Significance
C12:0	0.09^b^	0.07^b^	1.38^a^	0.18^b^	0.18	***
C14:0	2.13	2.07	2.27	2.29	0.23	NS
C14:1	0.56	0.55	0.56	0.53	0.08	NS
C15:0	0.57	0.50	0.54	0.59	0.04	NS
C15:1	0.36	0.41	0.22	0.35	0.16	NS
C16:0	19.82^b^	25.50^a^	21.14^b^	25.15^a^	0.57	***
C16:1	2.04	1.17	1.46	1.66	0.21	NS
C17:0	0.70	0.64	0.66	0.68	0.08	NS
C17:1	0.70	0.71	0.66	0.72	0.07	NS
C18:0	19.07^c^	24.53^a^	21.65^b^ ^c^	23.26^a^ ^b^	0.64	***
C18:1	31.74	31.57	32.13	32.46	1.19	NS
C18:1*trans*11	0.47	0.33	0.40	0.41	0.07	NS
C18:2 *n*-6	12.36^a^	9.38^b^	9.14^b^	9.04^b^	0.69	**
C18:3 *n*-3	0.60	0.63	0.61	0.69	0.05	NS
C20:0	0.39	0.37	0.38	0.38	0.01	NS
C20:1	0.39	0.35	0.41	0.36	0.05	NS
C20:3 *n*-6	0.66	0.61	0.55	0.61	0.05	NS
C20:4 *n*-6	5.30	4.52	4.46	4.37	0.28	NS
C24:1	0.67	0.62	0.60	0.63	0.04	NS
C20:5 *n*-3	0.39	0.32	0.32	0.39	0.05	NS
C22:5 *n*-3	0.56	0.63	0.58	0.62	0.04	NS
C22:6 *n*-3	0.41	0.32	0.36	0.36	0.4	NS
Σ SFA	43.89^b^	49.08^a^	49.78^a^	50.31^a^	0.88	***
Σ MUFA	35.17	36.27	36.42	37.12	1.05	NS
Σ *n*-3	1.95	1.89	1.89	2.06	0.1	NS
Σ *n*-6	18.32^a^	14.51^b^	14.16^b^	14.02^b^	0.82	**
Σ trans FA	0.47	0.33	0.40	0.41	0.07	NS
*n*-6/*n*-3	9.40^a^	7.75^a^ ^b^	7.55^a^ ^b^	6.90^b^	0.51	*
PUFA/SFA	0.46^a^	0.35^b^	0.34^b^	0.35^b^	0.03	*

CD, control diet; DCD, decanter cake diet; PKCD, palm kernel cake diet; CPOD, control+ 5% palm oil diet.

Σ SAF: Saturated fatty acid = sum of 12:0, 14:0, 15:0, 16:0, 17:0, 18:0, 20:0.

Σ MUFA: Monounsaturated fatty acid = sum of 14:1, 15:1, 16:1, 17:1, 18:1, 20:1, 24:1.

Σ *n*-3: Omega-3 fatty acid = sum of 18:3 n-3, 20:5 n-3, 22:5 n-3, 22:6 n-3.

Σ *n*-6: Omega-6 = sum of 18:2 n-6, C20:3 n-6, 20:4 n-6.

*n*-6/*n*-3: Σ *n*-6/Σ *n*-3.

PUFA: Polyunsaturated fatty acid

NS not significant (*P*>0.05).

* P<0.05

** P<0.01

*** P<0.001.

SE: standard error.

^a^ Means in the same row with different superscripts are significantly different.

^b^ Means in the same row with different superscripts are significantly different.

^c^ Means in the same row with different superscripts are significantly different.

The sum of SFA was lowest (*P*<0.05) and that of *n*-6 highest (*P*<0.05) for the CD than for the other diets. The ratio of *n*-6/*n*-3 was higher (*P*<0.05) for the CD than for the CPOD, but there were no differences among the CD, DCD and PKCD. The ratio of PUFA/SFA was higher (*P*<0.05) for the CD than for the other dietary treatments. The sums of MUFA, *n*-3 and trans FA were not different among the treatments.

### 3.2. Fatty acid composition of *biceps femoris*

The concentration of C12:0 in BF was higher (*P*<0.05) for the PKCD than for the CD, DCD and CPOD (Table 3), while the concentration of C18:1 was lower (*P*<0.05) and that of C18:2 *n*-6 was higher (*P*<0.05) for the CD than for the other dietary treatments. Concentrations of C16:0 and C18:0 were higher (*P*<0.05) for the CPOD and DCD compared to the CD while the differences between the PKCD and CD on one hand, and between the DCD and PKCD on the other hand, were not significant. The differences among the dietary treatments in the BF concentrations of other fatty acids were not significant.

**Table 3:** Effect of dietary treatments on the fatty acid composition (g/100g of total fatty acids) of the *biceps femoris* muscle of goats.

Fatty acids	CD	DCD	PKCD	CPOD	SE	significance
C12:0	0.19^b^	0.18^b^	1.22^a^	0.41^b^	0.19	**
C14:0	1.81	2.03	2.07	1.18	0.18	NS
C14:1	0.52	0.52	0.54	0.56	0.03	NS
C15:0	0.59	0.54	0.50	0.56	0.03	NS
C15:1	0.27	0.27	0.30	0.35	0.11	NS
C16:0	21.48^c^	26.02^a^ ^b^	23.83^b^ ^c^	27.58^a^	0.81	***
C16:1	2.28	2.27	2.64	2.69	0.21	NS
C17:0	0.71	0.64	0.62	0.65	0.07	NS
C17:1	0.70	0.62	0.65	0.69	0.05	NS
C18:0	20.03^c^	25.10^a^ ^b^	21.24^b^ ^c^	25.69^a^	0.92	**
C18:1	29.21^b^	32.58^a^	32.54^a^	32.68^a^	0.71	**
C18:1*trans*11	0.40	0.37	0.35	0.43	0.06	NS
C18:2 *n*-6	12.40 ^a^	9.26^b^	9.37^b^	9.54^b^	0.58	**
C18:3 *n*-3	0.64	0.61	0.59	0.64	0.04	NS
C20:0	0.39	0.39	0.37	0.38	0.02	NS
C20:1	0.47	0.39	0.41	0.38	0.05	NS
C20:3 *n*-6	0.65	0.56	0.62	0.58	0.04	NS
C20:4 *n*-6	5.19	4.61	4.49	4.09	0.29	NS
C24:1	0.68	0.60	0.53	0.60	0.04	NS
C20:5 *n*-3	0.52	0.49	0.49	0.51	0.01	NS
C22:5 *n*-3	0.61	0.57	0.53	0.60	0.03	NS
C22:6 *n*-3	0.44	0.34	0.35	0.35	0.05	NS
Σ SFA	45.18	46.69	46.85	46.20	1.08	NS
Σ MUFA	37.03	37.12	36.86	37.60	0.90	NS
Σ *n*-3	2.13	2.01	2.06	2.00	0.14	NS
Σ *n*-6	18.23^a^	14.43^b^	14.48^b^	14.21^b^	0.63	***
Σ trans FA	0.40	0.37	0.35	0.43	0.06	NS
*n*-6/*n*-3	9.68^a^	7.50^b^	7.41^b^	7.26^b^	0.47	***
PUFA/SFA	0.46^a^	0.35^b^	0.35^b^	0.35^b^	0.02	**

CD, control diet; DCD, decanter cake diet; PKCD, palm kernel cake diet; CPOD, control+ 5% palm oil diet.

Σ SAF: Saturated fatty acid = sum of 12:0, 14:0, 15:0, 16:0, 17:0, 18:0, 20:0.

Σ MUFA: Monounsaturated fatty acid = sum of 14:1, 15:1, 16:1, 17:1, 18:1, 20:1, 24:1.

Σ *n*-3: Omega-3 fatty acid sum of 18:3 n-3, 20:5 n-3, 22:5 n-3, 22:6 n-3.

Σ *n*-6: Omega-6 sum of 18:2 n-6, C20:3 n-6, 20:4 n-6.

*n*-6/*n*-3: Σ *n*-6/Σ *n*-3.

PUFA: Polyunsaturated fatty acid.

NS not significant (*P*>0.05)

** P<0.01

*** P<0.001

SE: standard error.

^a^ Means in the same row with different superscripts are significantly different.

^b^ Means in the same row with different superscripts are significantly different.

^c^ Means in the same row with different superscripts are significantly different.

There were no significant differences among the diets in the sum of SFA, MUFA, *n*-3 and trans FA in the BF, but the sum of *n*-6, *n*-6/*n*-3 ratio and PUFA/SFA ratio were all higher (*P*<0.05) for the CD than for the other treatments.

### 3.3. Fatty acid composition of *infraspinatus*

The concentration of C12:0 in the IS was higher (*P*<0.05) for the PKCD than for the CD, DCD and CPOD, while the concentration of C16:0 was higher (*P*<0.05) for both the DCD and CPOD than for the CD and PKCD (Table 4). The concentration of 18:0 was lower (*P*<0.05) for the CD than for the CPOD, but not different from the DCD and PKCD. The concentrations of C18:2 *n-6* and C20:4 *n*-6 were higher (*P*<0.05) for the CD than for the other treatments. There were no differences among treatments in the IS concentrations of the rest of individual fatty acids. There were no significant differences among treatments in the sums of MUFA, *n*-3 and trans FA in the IS, while the sum of *n*-6, *n*-6/*n*-3 ratio and PUFA/SFA ratio were all higher (*P*<0.05) for the CD than for the DCD, PKCD and CPOD.

**Table 4:** Effect of dietary treatments on the fatty acid composition (g/100g of total fatty acids) of the *infraspinatus* muscle of goats.

Fatty acids	CD	DCD	PKCD	CPOD	SE	significance
C12:0	0.31^b^	0.30^b^	1.56^a^	0.38^b^	0.16	***
C14:0	1.59	1.65	1.96	2.21	0.22	NS
C14:1	0.49	0.56	0.53	0.54	0.05	NS
C15:0	0.58	0.54	0.53	0.56	0.04	NS
C15:1	0.38	0.25	0.23	0.38	0.09	NS
C16:0	21.73^b^	26.56^a^	22.49^b^	26.29^a^	0.57	***
C16:1	1.51	1.89	1.88	1.80	0.21	NS
C17:0	0.63	0.65	0.53	0.65	0.08	NS
C17:1	0.66	0.59	0.59	0.62	0.05	NS
C18:0	21.74^b^	24.62^a^ ^b^	23.38^a^ ^b^	25.64^a^	0.86	*
C18:1	31.47	32.16	32.42	30.75	1.14	NS
C18:1*trans*11.	0.46	0.34	0.34	0.46	0.07	NS
C18:2 *n*-6	13.65^a^	10.07^b^	9.35^b^	9.52^b^	0.54	***
C18:3 *n*-3	0.61	0.62	0.65	0.66	0.04	NS
C20:0	0.40	0.38	0.40	0.38	0.02	NS
C20:1	0.39	0.38	0.34	0.37	0.03	NS
C20:3 *n*-6	0.63	0.56	0.55	0.57	0.04	NS
C20:4 *n*-6	5.64^a^	4.57^b^	4.48^b^	4.42^b^	0.20	**
C24:1	0.59	0.58	0.55	0.57	0.03	NS
C20:5 *n*-3	0.43	0.39	0.40	0.42	0.02	NS
C22:5 *n*-3	0.54	0.54	0.56	0.56	0.01	NS
C22:6 *n*-3	0.38	0.34	0.35	0.34	0.05	NS
Σ SFA	44.84^b^	46.36^a^ ^b^	46.91^a^ ^b^	48.70^a^	0.75	*
Σ MUFA	34.93	36.73	36.84	35.57	1.23	NS
Σ *n*-3	2.06	1.82	1.87	1.99	0.09	NS
Σ *n*-6	18.17^a^	14.34^b^	14.37^b^	14.49^b^	0.82	*
Σ trans FA	0.41	0.42	0.40	0.40	0.02	NS
*n*-6/*n*-3	9.43^a^	7.88^b^	7.73^b^	7.29^b^	0.29	***
PUFA/SFA	0.45^a^	0.37^b^	0.36^b^	0.35^b^	0.02	**

CD, control diet; DCD, decanter cake diet; PKCD, palm kernel cake diet; CPOD, control+ 5% palm oil diet.

Σ SAF: Total saturated fatty acid = sum of 12:0, 14:0, 15:0, 16:0, 17:0, 18:0, 20:0.

Σ MUFA: Total monounsaturated fatty acid = sum of 14:1, 15:1, 16:1, 17:1, 18:1, 20:1, 24:1.

Σ *n*-3: Omega-3 fatty acid = sum of 18:3 n-3, 20:5 n-3, 22:5 n-3, 22:6 n-3.

Σ *n*-6: Omega-6 = sum of 18:2 n-6, C20:3 n-6, 20:4 n-6.

*n*-6/*n*-3:Σn-6/Σn-3.

PUFA: Polyunsaturated fatty acid.

NS not significant (*P*>0.05).

*P<0.05

**P<0.01

***P<0.001

SE: standard error.

^a^ Means in the same row with different superscripts are significantly different.

^b^ Means in the same row with different superscripts are significantly different.

## Discussion

The treatment PKCD contained more C12:0 and C18:3 *n*-3 than the treatments CD, DCD and CPOD. The treatments DCD and CPOD were almost similar in their fatty acid profiles and both were rich in C16:0 and C18:1, probably because both DC and PO are generated from the mesocarp of the palm tree fruit. The highly saturated fatty acid composition found in PKC, and hence in the PKCD, is in line with the values reported by Cornelius [32] for palm kernel oil where the short chain fatty acid 12:0 is its main component. The most abundant fatty acids in all the muscles tested in the present study were C18:1, C16:0 and C18:0, which is in line with the fatty acid profile commonly reported for the goat meat [16,33–36]. Higher levels of C12:0 in PKC and consequently in the PKCD compared to the other diets translated in higher levels of this SFA in all the muscles from the goats fed the PKCD. Palm kernel cake fed lambs were reported to have more C12:0 and C14:0 SFA in their meat [20]. The increased proportion of C12:0 in meat of goats fed the PKCD in the present experiment can be of some concern as excessive consumption of medium chain SAF such as C12:0 raised the low-density lipoprotein cholesterol (LDL) concentrations in consumer’s blood tested for 8 weeks [37], thus, increasing the risk of the cardiovascular diseases. However, even though the constituent medium chain fatty acid in PKC may increase the total plasma cholesterol, a high proportion of this cholesterol is the beneficial high-density lipoprotein (HDL) [38]. An analysis in 60 human subjects concluded that C12:0 had a more favorable effect on the total/HDL cholesterol ratio than any other fatty acid studied [39].

The concentrations of C14:0, C14:1, C15:0, C15:1, C16:1, C17:0 and C17:1 in all the muscles were not significantly differed among the dietary treatments in the present experiment. However, on the average, the concentrations of C18:2 in all the muscles were always greater in the goats fed the CD than in those fed the other diets. The CD contained higher level of corn and thus more C18:2 than the other diets. Field *et al*. [40] and Bas and Morand-Fehr [19] reported that the C18:2 content of some fat depots increases when corn is the main source of the dietary energy. The PUFA present in corn are more resistant to biohydrogenation than the PUFA in other cereals [41] suggesting that more of these fatty acids reach fat depots. Moreover, the complete biohydrogenation of C18:2 to C18:0 might be inhibited by the presence of large amounts of C18:2 [42]. The incomplete hydrogenation might be the reason behind the slightly lower C18:0 concentrations in the muscles of goats fed the CD as compared to the other diets.

Relatively higher proportion of total SFA in the LD of goats fed the DCD and PKCD in comparison to those fed the CD in the present experiment might be a reflection of the higher concentration of SFA in the former diets. This is in line with Solomon *et al*. [43] who reported that ram and ewe lambs fed PO supplemented diets produced more intramuscular SFA. The authors suggested that the increased SFA could be due to the increase in palmitic acid. In the present experiment, however, the palmitic acid (C16:0) proportions were slightly higher for the DCD than for the CD. The observed higher proportion of SFA is probably due to the higher dietary fiber intake for the DCD and PKCD as the fiber contents of these diets is higher than those in the CD and CPOD. The dietary fiber fraction is known to increase rumen activity and consequently increasing the extent of biohydrogenation of the dietary PUFA by the rumen microbes [44,45]. In line with the findings in the present experiment, Boer × Spanish male goats [35] and East African goats [46] fed hay alone had higher SFA in subcutaneous fat and minced meat, respectively when compared to goats fed concentrate diets.

The current recommendations are that PUFA/SFA ratios should be around 0.45 [47]. In the present experiment, however, the ratios remained below the recommended value in all the muscles of goats fed the DCD, PKCD and CPOD than of those fed the CD. Nevertheless, lower values were reported in the feral goats in Australia [48] and Pateri goats in Pakistan [16] under different nutritional managements. Such lower values have been related to hydrogenation of dietary unsaturated fat in the rumen [49] which in turn results in a lower PUFA/SFA ratio in the meat of ruminants. The ratio of PUFA *n*-6/ PUFA *n*-3 was higher in all the muscles of goats fed the CD than of those fed the CPOD. This might be due to a relatively higher C18:2 *n*-6, C20:3 *n*-6 and C20:4 *n*-6 in the muscles of goats fed the CD. The values of *n*-6/ *n*-3 ratio obtained in the present experiment is higher than the value of 4 or less recommended by the HMSO [50], however, lower [16,51–52] and higher [53] values were reported in different ruminant meat in different experiments. The inconsistent data could be related to different dietary management in different experiments according to De Smet *et al*. [54]. These authors postulated that *n*-6/*n*-3 ratios are much more affected by the feeding management rather than by the genetics of the animal.

*Longissimus dorsi* was used as a reference muscle in most published work studying the fatty acid profile of the intramuscular fat. However, Barton *et al*. [55], Pena *et al*. [4] and Talpur *et al*. [16] noted significant differences in the fatty acid profile between different muscles. In contrast, Costa *et al*. [56] found no differences in the SFA and unsaturated fatty acid contents between the *semitendinosus* and LD muscles in young bulls. In the present experiment, almost similar fatty acid profiles were observed in all the muscles.

## Conclusion

Palm kernel cake and DC are low cost easily available feed resources in Malaysia that could be included in diets of goats up to the presently used 80% level with more beneficial than detrimental effects on the fatty acid profile of meat. However, higher levels could change the fatty acid profile of the chevon meat towards saturation, which is not favorable to the health conscious consumers.
